# Crystal structure of the *Yersinia enterocolitica* type III secretion chaperone SycD in complex with a peptide of the minor translocator YopD

**DOI:** 10.1186/1472-6807-12-13

**Published:** 2012-06-18

**Authors:** Madeleine Schreiner, Hartmut H Niemann

**Affiliations:** 1 Department of Chemistry, Bielefeld University, PO Box 10 01 31, 33501, Bielefeld, Germany

**Keywords:** Bacterial virulence factor, Chaperone, Complex, Crystal structure, Dimer, Peptide binding, Protein-protein interaction, Tetratricopeptide repeat, Translocator, Type III secretion

## Abstract

**Background:**

Type III secretion systems are used by Gram-negative bacteria as “macromolecular syringes” to inject effector proteins into eukaryotic cells. Two hydrophobic proteins called translocators form the necessary pore in the host cell membrane. Both translocators depend on binding to a single chaperone in the bacterial cytoplasm to ensure their stability and efficient transport through the secretion needle. It was suggested that the conserved chaperones bind the more divergent translocators via a hexapeptide motif that is found in both translocators and conserved between species.

**Results:**

We crystallized a synthetic decapeptide from the *Yersinia enterocolitica* minor type III secretion translocator YopD bound to its cognate chaperone SycD and determined the complex structure at 2.5 Å resolution. The structure of peptide-bound SycD is almost identical to that of apo SycD with an all helical fold consisting of three tetratricopeptide repeats (TPRs) and an additional C-terminal helix. Peptide-bound SycD formed a kinked head-to-head dimer that had previously been observed for the apo form of SycD. The homodimer interface comprises both helices of the first tetratricopeptide repeat. The YopD peptide bound in extended conformation into a mainly hydrophobic groove on the concave side of SycD. TPRs 1 and 2 of SycD form three hydrophobic pockets that accommodated the conserved hydrophobic residues at position 1, 3 and 6 of the translocator hexapeptide sequence. Two tyrosines that are highly conserved among translocator chaperones contribute to the hydrophobic patches but also form hydrogen bonds to the peptide backbone.

**Conclusions:**

The interaction between SycD and YopD is very similar to the binding of the *Pseudomonas* minor translocator PopD to its chaperone PcrH and the *Shigella* major translocator IpaB to its chaperone IpgC. This confirms the prediction made by Kolbe and co-workers that a hexapeptide with hydrophobic residues at three positions is a conserved chaperone binding motif. Because the hydrophobic groove on the concave side of translocator chaperones is involved in binding of the major and the minor translocator, simultaneous binding of both translocators to a single type III secretion class II chaperone appears unlikely.

## Background

A wide range of pathogenic Gram-negative bacteria use a type III secretion system (T3SS) to facilitate the transport of cytotoxins, so called effector proteins, into the host cell. These effectors influence and manipulate diverse cellular pathways for the pathogens’ benefit resulting e.g. in a repression of the inflammatory response or altered phagocytosis [1,2]. This allows the bacteria to thrive undetected by the host’s immune system.

Effector translocation occurs via the injectisome, a needle-like multi-protein complex evolutionarily related to the bacterial flagellum. The injectisome’s basal body spans the whole bacterial envelope eventually narrowing into a hollow needle protruding from the bacterial surface. The effectors pass the host cell membrane through pores formed by translocator proteins arranging in a ring like structure (for detailed reviews see [3-6]). The ring forming translocator proteins are sub-divided in the major and the minor translocator. Though both proteins exhibit a hydrophobic nature and exist in oligomeric states, they share almost no sequence homology. The major translocator holds an N-terminal coiled-coil domain, the crystal structure of which was recently reported [7], and two predicted transmembrane helices as common and conserved features between species. Major translocators have been shown to insert into membranes even in absence of the hydrophobic binding partner [8-10]. Nevertheless the minor translocator is essential for the formation of a functional pore [10,11]. The minor translocator only features one predicted transmembrane region, a coiled-coil domain and a C-terminal amphipathic region, which is important for the binding to the hydrophilic translocator (LcrV in *Yersinia spp.*). The proteins are well conserved between species and have been shown to exist in a partly unfolded and disordered state that seems to be necessary for translocation and pore formation (for detailed review see [12]). In some cases the minor translocator is additionally involved in regulatory processes [13].

For efficient export both hydrophobic translocators and several effectors need specific chaperones that stabilize them in the bacterial cytosol prior to export. In contrast to classic chaperones like DnaK or GroEL these proteins lack the ability to bind and hydrolyze ATP. T3S chaperones have been divided into three subgroups: Class I chaperones interacting with effector proteins, class II chaperones interacting with the translocators and class III chaperones interacting with T3SS needle components (for detailed reviews see [14-16]). T3S chaperones prevent premature aggregation and keep their substrates in a partially unfolded state so that they are able to pass the narrow channel of the secretion needle. A hexameric ATPase associated to the cytoplasmic side of the T3S export apparatus powers chaperone release and further unfolding of effectors [17,18]. Deletion of the hexameric ATPase was also shown to impair secretion of a translocon protein [19].

SycD (specific Yop chaperone D) from the enteropathogen *Yersinia enterocolitica* is the class II chaperone of the translocator proteins YopD (*Yersinia* outer protein D) and YopB (*Yersinia* outer protein B) [20]. Like all known class II chaperones SycD exhibits an all α-helical structure [21]. Six helices (H1A, H1B, H2A, H2B, H3A, H3B) fold into three tetratricopeptide repeats (TPRs), tandemly arranged motifs of some 34 amino acids length [22]. An extra C-terminal helix (H8) probably exerts a stabilizing function. TPRs generally provide platforms for protein-protein interactions. They are e.g. found in one protein of the heterodimeric chaperones for the T3SS needle proteins [23-25]. The superhelical arrangement of tandem TPRs results in a curved structure with a convex outer surface and a concave inner side, which forms a mostly hydrophobic groove in T3S class II chaperones. Crystal structures of SycD homologs in complex with peptides from their cognate translocator proteins have shown that the concave surface provides a binding site for the translocator proteins. Recently, Lunelli *et al.* identified a short binding motif (^65^PELKAP^70^) within the *Shigella* major translocator IpaB that binds into the concave groove of its chaperone IpgC [26]. Based on these results they further suggested that similar motifs in other translocators including the *Yersinia* proteins YopD and YopB are also involved in chaperone binding. Subsequently Dessen and co-workers revealed that the concave groove of the *Pseudomonas* chaperone PcrH constitutes the binding platform for the minor translocator PopD sequence ^49^VELNAP^54^ showing high similarity to the IpgC:IpaB binding motif [27].

Here we present the structure of the class II chaperone SycD in complex with a peptide of the N-terminal region of the minor translocator YopD comprising the recently proposed sequence motif PELIKP. The peptide binds in an elongated form into the chaperone’s concave groove where three highly conserved hydrophobic residues point into three conserved hydrophobic pockets formed by the chaperone. These results underline that class II T3S chaperones recognize the major and the minor translocator via a common sequence motif that is present in a wide range of T3S translocator proteins.

## Results

### Peptide-bound SycD crystallized as a kinked head-to-head dimer

In order to verify that YopD binds to SycD via the proposed sequence motif ^58^PELIKP^63^[26] we co-crystallized N- and C-terminally truncated SycD_21-163_ with a synthetic peptide corresponding to YopD_56-65_ (^56^QVPELIKPSQ^65^). The chaperone peptide complex crystallized in the trigonal space group P3_1_21 with one monomer per asymmetric unit. The structure was solved to a resolution of 2.5 Å by molecular replacement using SycD_21-163_ (PDB-ID: 2VGY) as search model. Clearly visible Fo-Fc difference electron density within the concave groove revealed that the peptide had been successfully co-crystallized with SycD_21-163_. Data collection and refinement statistics are listed in Table 1.

**Table 1:** X-ray data-collection and refinement statistics

	**SycD**_**21-163**_**/YopD**
**Data collection**	
Wavelength (Å)	0.918
Space group	*P*3_1_21
Unit cell parameters	
a, b, c (Å)	106.4, 106.4, 52.0
α, β, γ (°)	90, 90, 120
Resolution range (Å)	25–2.5 (2.64-2.5)
No. observed/unique reflections	166476/11931
Completeness	99.4 (97.0)
Multiplicity	14.0 (10.0)
R_*merge*_ (%)^*^	10.6 (57.1)
Mean *I/σ(I)*	18.5 (3.1)
Wilson B factor (Å²)	79.6
**Refinement**	
R_*work*_/R_*free*_ (%)^§^	19.0/23.8 (31.3/34.8)
Mean B factor (Å²)	
Overall	79.2
Protein	78.1
Peptide	84.0
Ligand/ion	105.3
Water	75.3
No. of atoms	
Protein/peptide	1127
Ligand/ion	35
Water	17
R.m.s.d.	
bond (Å)	0.007
angle (°)	1.061
Ramachandran	
favored (%)	95.6
allowed (%)	4.4

$*{\text{R}}_{\mathrm{merge}}=\sum_{\mathrm{hkl}}\sum_{i}|{\text{I}}_{\text{i}}(\text{h}\text{k}\text{l})-\text{I}\langle (\text{h}\text{k}\text{l})\rangle |/\sum_{\mathrm{hkl}}\sum_{i}{\text{I}}_{\text{i}}(hkl)$ where I_*i*_(hkl) is the *i*th measurement of the reflection (hkl).

^§^${\text{R}}_{\mathrm{work}}/{\text{R}}_{\mathrm{free}}=\sum_{\mathrm{hkl}}\left||{\text{F}}_{\mathrm{obs}}|-|{\text{F}}_{\mathrm{calc}}|\right|/\sum_{\mathrm{hkl}}||{\text{F}}_{\mathrm{obs}}||$. R_*work*_ was calculated from the work set. R_*free*_ was calculated from the test set encompassing 4.9% of the total reflections. The test set was not used in refinement. Values in parentheses belong to the highest resolution shell.

**Figure 1:**
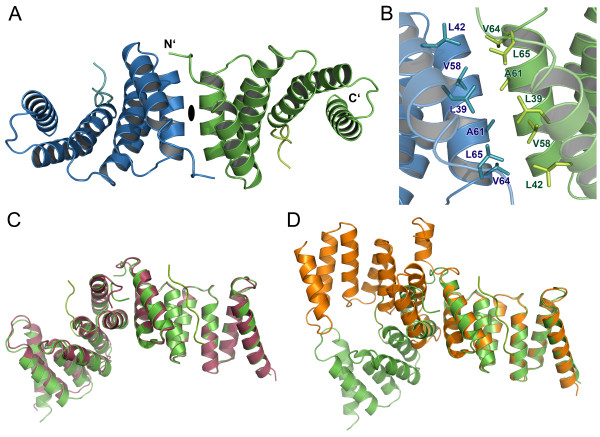
**Dimer arrangement of the SycD:YopD complex. (A)** Peptide-bound SycD forms a symmetric dimer, in which protomers are related by a crystallographic 2-fold axis. The dimerization is mediated via the two N-terminal helices (TPR1). **(B)** Dimerization interface of the SycD:YopD complex stabilized by van der Waal contacts involving the labeled residues shown as sticks. **(C)** Pairwise alignment (DaliLite server [29]) of SycD:YopD (green) to kinked apo SycD (red) (PDB ID: 2VGY) and to **(D)** monomer B of elongated apo SycD (orange) (PDB ID: 2VGX) shows that the dimer arrangement of the chaperone-peptide complex is nearly identical to the arrangement of the kinked apo form.

The crystal packing of the peptide-bound variant of SycD reveals a 2-fold symmetric dimer (Figure 1A) that is very similar to the alternative kinked dimer of apo SycD_21-163_ (PDB-ID: 2VGY) (Figure 1C, D), one of two possible elongated dimers previously described by Büttner *et al.*[21]. The dimerization site consists of both helices of the first tetratricopeptide repeat (TPR1) (Figure 1B) with a buried surface area of 710 Å² per monomer as defined by the PISA server [28] and mainly involves the hydrophobic residues Leu42, Val58, Ala61 and Leu65. The residues Leu65 and Ala61 have previously been shown to be essential for SycD dimer formation [21].

A pairwise comparison done by the DALI Lite server [29] showed that the SycD protomer in the crystal structure of the SycD_21-163_:YopD complex is structurally very similar to the SycD_21-163_ protomer from crystal form 2 (kinked dimer, PDB-ID: 2VGY) with a root mean square deviation (r.m.s.d.) of 0.8 Å and to both chains of SycD_21-163_ crystal form 1 (elongated dimer, PDB-ID: 2VGX) with an r.m.s.d. of 0.9 Å for monomer A and an r.m.s.d. of 1.2 Å for monomer B for Cα-atoms. The residues lining the concave groove do not show significant conformational changes between the apo and the peptide-bound form, suggesting a preformed and largely rigid binding site. Yet small differences exist with regard to the position and angle of the stabilizing helix H8 suggesting that it is somewhat flexible. However, except for residue Arg146 helix H8 does not contribute to peptide binding. Arg146 seems to be relatively flexible in the SycD apo forms but is fixed in the complex structure via hydrogen bonds to Glu142 and the peptide residue Gln56’ (apostrophe further denotes YopD peptide residues). The ordering of Arg146 in the complex is partly also brought about by contacts to Gln131 and Phe128 of a neighboring monomer, and may thus be due to crystal packing rather than peptide binding.

### The YopD sequence motif is recognized by the concave groove of SycD

**Figure 2:**
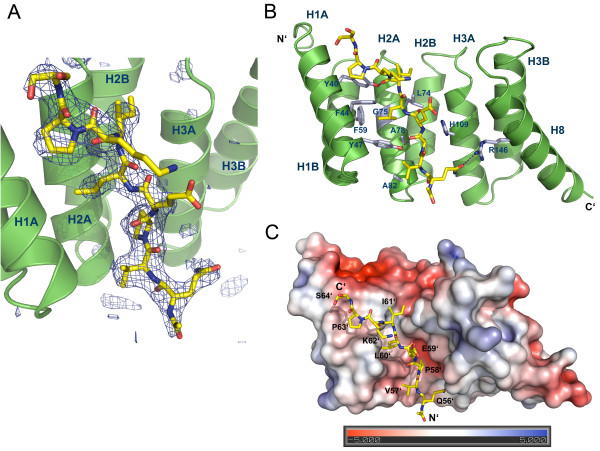
**Structure of SycD in complex with the YopD peptide. (A)** Positive difference electron density within the Fo-Fc map contoured at 3σ (blue mesh) assigns the position of the YopD peptide (yellow) to the concave groove of SycD (green). The Fo-Fc map was calculated via simulated annealing refinement in PHENIX [30] using the fully refined structural model whose YopD peptide chain was removed prior to refinement. **(B)** The YopD peptide lies antiparallel to helix H1A within the concave cleft interacting with various mainly hydrophobic residues lining H1A, H2A and H3A. Hydrogen bonds (purple lines) involving Tyr40, Tyr47 and Arg146 additionally stabilize the complex. **(C)** Electrostatic surface potential of the SycD:YopD complex. Three nonpolar residues Pro58’, Pro63’ and Leu60’ occupy distinct hydrophobic pockets and anchor the peptide (yellow) into the concave cleft. Negatively charged surface areas are colored in red, positively charged areas in blue.

The Fo-Fc difference map clearly revealed positive electron density within the concave groove of SycD_21-163_ (Figure 2A), which allowed the easy positioning of the peptide residues 56’-64’ whereas Gln65’ could not be placed due to missing electron density. The peptide binds in an extended form into the chaperone’s hydrophobic cleft that is lined by aromatic and aliphatic residues from helix H1A and H2A (Figure 2B). The antiparallel orientation relative to helix H1A allows the peptide’s hydrophobic residues Pro58’, Leu60’ and Pro63’ to perfectly fit into three distinct hydrophobic pockets (Figure 2C) within the concave groove whereas the charged residues Glu59’ and Lys62’ point outwards without contacting the chaperone. The pocket for Leu60’ of the peptide is exclusively hydrophobic and is mainly built by the SycD residues Phe44, Tyr47 (H1A), Phe59 (H1B), Leu74, Gly75 and Ala78 (H2A) (Figure 2B). Pro63’ resides on a smaller hydrophobic patch consisting of Tyr40 and Phe44 (H1A). The region of the concave groove that binds Pro58’ has a mixed hydrophobic and polar character and is formed by Tyr47 (H1A), Ala78 (H2A), Gln81 (H2A), Tyr93 (H2B) and His109 (H3A). Van der Waals contacts between Tyr52 and Ala82 from SycD and Val57’ of the peptide and between Leu74 of SycD and Ile61’ of the peptide further support the complex. Direct hydrogen bonds are formed between the hydroxyls of SycD Tyr40 and Tyr47 and the carbonyl oxygen of the peptide residues Ile61’ and Pro58’, respectively, and between the guanidino group of Arg146 (H8) and the side chain of Gln56’. In addition, there are several water mediated interactions between SycD and the YopD peptide.

**Figure 3:**
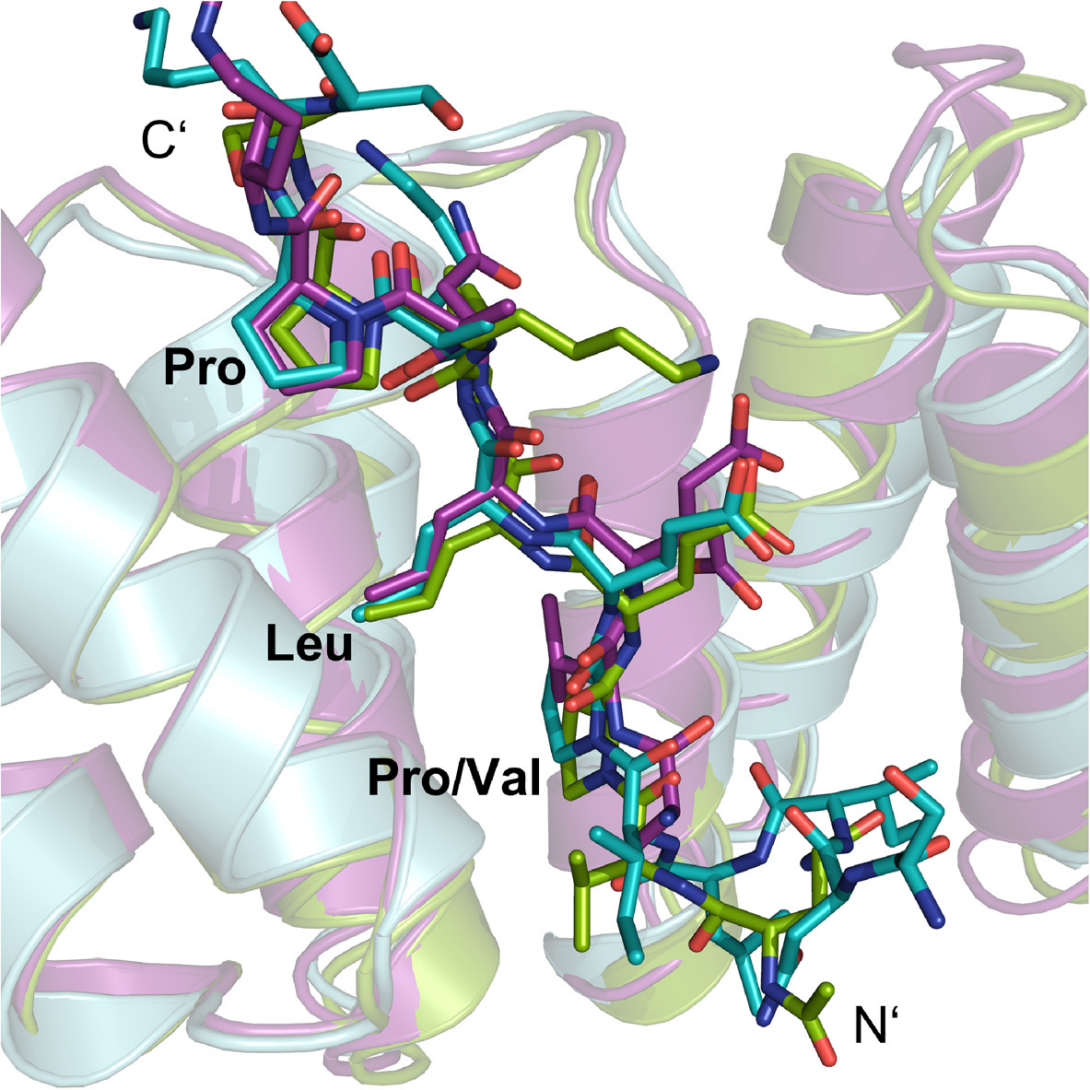
**Superposition of the chaperone binding domains of T3S translocators.** The superposition (DaliLite server [29]) of the chaperone-peptide complexes SycD:YopD (green), PcrH:PopD (purple) (PDB ID: 2XCB, chain A) and IpgC:IpaB (cyan) (PDB ID: 3GZ1, chain B) reveals a good spatial agreement within the key anchor residues of the YopD, PopD and IpaB sequence motifs.

Structure based sequence alignments (see [27]) revealed that the chaperone binding domain (CBD) harbors three evolutionary highly conserved residues, which have been identified to be essential for the PcrH:PopD and IpgC:IpaB complex formation [26,27]. These are interspaced by unspecific, mostly hydrophilic residues that may merely function as spacers leading to the common motif P/VxLxxP, where x is either hydrophilic or hydrophobic. Based on these results it is evident that YopD residues Pro58’, Leu60’ and Pro63’ correspond to IpaB Pro65, Leu67 and Pro70 and to PopD Val49, Leu51 and Pro54. Furthermore a superposition of the chaperone Cα-atoms of SycD:YopD with PcrH:PopD (PDB-ID: 2XCB, chain A) and IpgC:IpaB (PDB-ID: 3GZ1, chain B) with an r.m.s.d. of 1.0 Å and 1.2 Å, respectively, showed that the minor translocator YopD binds SycD in a similar fashion (Figure 3). In all three chaperones the bound peptide fragments adopt a nearly identical conformation within the main sequence motif leading to a very good overlap of the three hydrophobic key residues that occupy the same hydrophobic patches.

## Discussion

One unexpected finding of this work is the SycD quaternary structure. We had previously shown that mutations in helix H1B result in monomeric SycD, thereby mapping the homodimerization interface to TPR1 [21]. However, the precise quaternary structure remained ambiguous, because two different crystal forms revealed different arrangements of the protomers in the SycD dimer, an extended and a kinked one [21]. The elongated dimer (PDB ID: 2VGX) had a larger buried surface area, a better shape correlation and a smaller gap volume index, suggesting that it is more likely to represent the solution dimer. Surprisingly, the structure reported here contains a SycD homodimer that is virtually identical to the kinked SycD dimer reported previously (PDB ID: 2VGY). Crystals of the SycD:YopD complex and the kinked apo form of SycD are not isomorphous and in different space groups. This suggests that the kinked dimer may be more favorable than initially thought [31].

Ambiguity and variability of their quaternary structures appears to be a recurring theme among T3S translocator chaperones. Formation of higher oligomers has been reported for full-length SycD [32] or *Salmonella enterica* SicA [33]. It is of note that homodimers of T3S class II chaperones are not constitutive. PcrH shows monomer-dimer equilibrium [27]. Moreover, binding of translocators often disrupts the dimer interaction, resulting in 1:1 complexes [27,34,35], although binding of a single translocator to a dimeric chaperone has also been reported [36]. The arrangement of protomers in the chaperone dimers is under debate in several cases as well. For IpgC two different asymmetric dimers [26] and another 2-fold symmetric dimer involving TPR1 [37] were reported. The crystal structure of PcrH shows two quaternary structures that might be stable in solution, an asymmetric dimer mediated by contacts of the convex side (back-to-back dimer) and a 2-fold symmetric dimer mediated by TPR1 (head-to-head) [27]. For PcrH, no functional data is available to decide which of these dimers is present in solution.

Previous studies had led to the identification of two regions within YopD that are essential for SycD binding [38], one involving the C-terminal amphipathic domain (aa 278–292) and another one located N-terminally encompassing the residues 53–149, which also contains the six amino acid consensus sequence for binding to class II chaperones identified by Kolbe and co-workers [26]. The concave groove of TPR proteins can accommodate interaction partners in helical [24,25,39,40] or extended conformation [41-43]. Accordingly the SycD concave groove was proposed to bind the amphipathic YopD C-terminus in helical conformation [44] or a short conserved peptide from the N-terminal region in extended conformation [26]. Our structure verifies that the YopD consensus peptide binds to SycD in the manner predicted by Kolbe and co-workers based on their IpgC:IpaB complex structure [26]. The peptide’s three conserved anchor residues bind into distinct binding pockets within the concave region leading to a peptide conformation that is nearly identical to that of the CBDs of PopD and IpaB in complex with their respective chaperones [26,27]. This allows understanding how TPR-like chaperones that are well conserved between species (sequence identity SycD/PcrH: 59%, SycD/IpgC: 28%, (see also [21,26])) bind to their respective cargo that exhibits a lower degree of sequence conservation (sequence identity (1) minor translocator: YopD/PopD:41%, YopD/IpaC: 20%; (2) major translocator YopB/PopB: 43%, YopB/IpaB: 17% (see also [12,27])) and how they recognize with high specificity two different substrates that share nearly no common sequence features. The presence of the consensus motif for binding of the chaperone’s hydrophobic groove in both the major and the minor translocator from the same species suggests that binding of translocators to the chaperone is mutually exclusive and formation of a ternary complex should not be possible. The data in the literature with regard to this issue is not univocal and the situation may well differ between species [27,35,36,45,46]. In some cases, e.g. in *Edwardsiella tarda*, each hydrophobic translocator even requires its own chaperone [47,48].

The hydrophobic cleft of SycD is lined by aromatic residues, mainly tyrosines, which have been shown to appear with high prevalence in a wide range of protein interaction surfaces [49]. These residues are highly conserved within T3S class II chaperones although they are located at non-canonical positions [21,34,44]. Tyr40 and Tyr47 are not only involved in the formation of hydrophobic patches but also keep the peptide in the correct conformation by providing a functional group for hydrogen bond formation with the peptide backbone. The existence of very similar interactions in the chaperone-peptide complexes of IpgC from *Shigella*[26] and PcrH from *Pseudomonas*[27] underlines the importance hereof. Thus it is not surprising, that mutations within the conserved aromatic ladder of helix H1A (Tyr40, Phe44, Tyr47 in SycD) have severe effects on the recognition and binding of YopD and YopB [50]. Based on the present structure it is not possible to rationalize why several mutations that map to canonical TPR positions or the binding groove should affect binding and/or secretion only of YopB but not of YopD [50]. Mutations affecting secretion only of YopD but not YopB were mapped to the convex side (L42A, H67A and L76A), which thus was considered to be SycD’s main binding region for YopD, with additional contributions from the loop connecting H2B and H3A [38,50,51]. These observations may be explained by the fact that the CBD represents only a small part of a longer N-terminal binding region that together with the C-terminal amphipathic domain is involved in SycD binding. Furthermore, the residual part (aa 150–287) of the minor translocator has been shown to exist in a partially unfolded molten globule state [52]. Thus one might assume that YopD and most likely YopB are recognized by the chaperone’s concave cleft through the conserved binding motif. The partly unfolded region of the protein could wrap around SycD so that YopD might contact the convex side with the remaining binding regions.

## Conclusions

Compared to the effector binding chaperones of T3SSs still only little is now about the TPR-like class II chaperones especially concerning the chaperone translocator interaction. This is mainly due to the hydrophobic nature and thus delicate handling of the translocator proteins. By co-crystallizing SycD with a synthetic peptide, we were able to show that the concave side of SycD interacts with a conserved chaperone binding hexapeptide of the minor translocator YopD. These results underline the fact that T3S TPR-like chaperones bind their substrates via a common mechanism that is highly conserved between different species. However, several questions remain. The exact quaternary structure in solution is currently not established for any of the T3S translocator chaperones for which crystal structures are available. Complementary techniques like solution small angle scattering may help to resolve this issue. Moreover, the current structures of chaperones bound to short translocator peptides do not explain why binding of substrates causes dissociation of the chaperone homodimers and results in heterodimeric 1:1 complexes in many cases. Thus, T3S translocators, their chaperones and the complexes formed between them remain an exciting topic for structural studies.

## Methods

### Protein expression and purification

Expression of glutathione-S-transferase (GST)-tagged SycD_21-163_ was performed as described [21] in *E. coli* BL21 (DE3) RIL transformed with the expression vector pGEX-6P-1_*syc*D21-163. Bacteria were grown in LB medium at 37°C to OD_600_ ~ 0.4-0.5. The temperature was reduced to 20°C and incubation was continued to OD_600_ ~ 0.8. Protein expression was induced by adding 0.25 mM isopropyl-ß-d-thio-galactopyranoside (IPTG) and was carried out for 16–18 h at 20°C. Pelleted cells were resuspended in phosphate buffered saline (PBS) containing 10 mM ß-mercaptoethanol, 1 tablet Complete protease inhibitor cocktail (Roche) and DNase and lysed using a French pressure cell press. The lysate was centrifuged at 16'000 xg. The supernatant was added to a glutathione sepharose matrix equilibrated with PBS pH 7.4, 10 mM β-mercaptoethanol and incubated for 1 h at 4°C. The column was washed extensively with PBS and with 2–3 column volumes 50 mM Tris pH 7.4, 200 mM NaCl, 0.5 mM EDTA, 10 mM β-mercaptoethanol. The GST-tag was cleaved off with PreScission Protease in 2–3 column volumes of the same buffer containing 40 μM Pefabloc and 0.3 μM aprotinin for ~ 48–66 h at 4°C. The cleaved protein was eluted and dialysed against 50 mM Tris pH 8, 4 mM dithiothreitol (DTT). SycD_21-163_ was further purified via anion exchange chromatography (Source 15Q, GE Healthcare) using a linear NaCl gradient with 50 mM Tris pH 8, 4 mM DTT, 1 M NaCl as high salt buffer followed by size exclusion chromatography (Superdex 75 16/60, GE Healthcare) equilibrated with 20 mM Tris pH 8, 50 mM NaCl, 1 mM DTT. The protein was concentrated to ~ 15 mg/ml and stored at −80°C until further use.

### Crystallization

A synthetic peptide corresponding to YopD_56-65_ (Ac-QVPELIKPSQ-NH_2_) was purchased from EMC microcollections (Tübingen). For co-crystallization experiments the YopD peptide was dissolved in 20 mM Tris pH 8, 50 mM NaCl, 1 mM DTT with a concentration of 6 mM and mixed with SycD_21-163_ in the same buffer in a molar ratio of 1:1.3 (SycD:Peptide). Initial crystallization conditions were found using commercially available screens (Qiagen). Final crystals were grown at 20°C using the vapor diffusion method in optimized conditions containing 50 mM MES pH 6, 50 mM citrate pH 5, 1.1-1.4 M (NH_4_)_2_SO_4_, a drop ratio of 1:0.5 protein:reservoir and a protein concentration of 8 mg/mL. Single crystals were harvested, cryoprotected with 15% 2,3-butanediol in mother liquor and flash frozen in liquid nitrogen.

### Data collection, structure determination and refinement

Diffraction data were collected at 100 K and a wavelength of 0.918 Å at Beamline 14.2 at BESSY II (Berlin) on a Rayonics MX-225 CCD detector with an oscillation range of 0.3° or 0.5°. Data were indexed and integrated with XDS [53], merged in POINTLESS and scaled with SCALA [54] from the CCP4 suite [55]. Phases were obtained via molecular replacement in PHASER [56] using SycD_21-163_ (PDB-ID: 2VGY) as search model. Model building was carried out in COOT 0.6.2 [57] and the structure was refined with PHENIX.REFINE 1.7.2 [30]. The structure was validated using the MolProbity server [58] and the validation tools implemented in COOT. The YopD peptide was built and refined with an occupancy of 1.0. This approach was validated by a final refinement, in which the starting occupancy of the YopD peptide was set to 0.5 and a single group occupancy was refined for the whole peptide, resulting in a refined occupancy of 0.97. Using the output model as input for a second round of refinement led to an occupancy of 1.0 for the YopD peptide. For the calculation of a weighted 2Fo-Fc and Fo-Fc map showing the position of the peptide ligand, the YopD peptide chain was removed from the fully refined model prior to ten macrocycles of simulated annealing refinement in PHENIX. Figures were created with PyMOL [59]. The electrostatic surface potential of the SycD_21-163_:YopD_56-65_ complex was calculated with the APBS [60] plug-in of PyMOL using default settings.

## Abbreviations

CBD, Chaperone binding domain; DTT, Dithiothreitol; GST, Glutathione-S-transferase; Syc, Specific yop chaperone; T3SS, Type III secretion system; TPR, Tetratricopeptide repeat; Yop, Yersinia outer protein.

## Competing interests

The authors declare that they have no competing interests.

## Authors’ contributions

HHN conceived the study. MS purified protein, processed data, and solved and refined the structure. MS and HHN designed experiments, collected diffraction data, evaluated results and wrote the manuscript. All authors read and approved the final manuscript.
